# Who goes where? The importance of peer groups on attainment and the student use of the lecture theatre teaching space

**DOI:** 10.1002/2211-5463.12494

**Published:** 2018-08-21

**Authors:** David P. Smith, Angela Hoare, Melissa M. Lacey

**Affiliations:** ^1^ Department of Biosciences and Chemistry Sheffield Hallam University UK

**Keywords:** engagement, learning space, lecture, peer groups, performance, seat location

## Abstract

Understanding how students interact and learn within the lecture theatre environment is central to successful learning outcomes. Previous studies into the use of the lecture theatre teaching space have found that students sit in specific locations due to a range of factors; these include being noticed, addressing anxiety or an ability to focus. This study further explores the personal and social factors at play within students’ lecture theatre seating choice and the resulting effects on attainment. Student responses on seating preferences detailing why they chose a given location were mapped at a seat‐specific level and correlated against attainment. In parallel, staff perceptions of student attainment in relation to their seating choice were obtained. No direct correlation between student location and attainment was found, contrary to staff perceptions. Interestingly, it was found that students physically locate into friendship groups clusters and that these clusters obtained similar levels of attainment in problem‐solving tasks, with pockets of both high‐ and low‐performing students being observed. It was also noted that isolated students performed less well. These data would indicate that peer group formation exerts a strong impact on attainment and engagement. Outcomes from this study will enable academic staff to better understand the student body and inform the way in which teaching sessions are performed within a lecture theatre.

The history of teaching through lectures and higher education are interwoven, with many viewpoints and discussions about their effectiveness being presented. The increase in student numbers and pressures on teaching budgets mean they remain the most pragmatic approach to teaching content‐rich material to medium‐to‐large student groups. As such, a large proportion of university teaching occurs in this environment, which indicates the need for ongoing research into the use of this space by both staff and students. Here, we investigate the factors effecting seating choice within a lecture theatre and the underlying factors that govern student behaviour and attainment within this learning space.

## The lecturing environment and factors affecting academic success

Early studies undertaken into student interactions in the lecture theatre found that students who sat within the middle of a row contributed more frequently in discussions than students sat at the edge 1 and students in the front and centre of a lecture theatre communicated more with the teacher. In addition, students located at the front rated themselves as more intelligent and liked by the teacher compared to those who chose to sit at the back 2. Holliman and Anderson 3 enhanced the field by analysing attainment by row, they found students on rows near the front performed better in examinations, whereas location on a specific row had no effect on grades. This is supported by Marshall and Losonczy‐Marshal 4 who upon completion of one of the longest running studies spanning 15 years and collecting data from over 70 classes found students in a central location attended lectures more often and also performed better in projects and examinations. It is, however, worth noting that these finding are not ubiquitous, as Kalinowski and Taper 5 using similar methods to that of Perkins and Wieman 6 found seating location had no effect on the student's grades or attitudes.

To a lesser extent, the effect of the student's personality on seating location and subsequent attainment has been researched within the field. Stires 7 found students who sat in a central location achieved the highest grades in examinations both if they were randomly allocated a central seat and if they independently chose to sit centrally, thus implying the ecology of the lecture theatre has a greater impact on attainment than the students’ personality. However, multiple studies found that individuals located towards the front of the class self‐reported higher levels of motivation, with those towards the back stating they want to avoid interaction 8, 9. The juxtaposition within the literature is combined in part by Perkins and Wieman 6 who found when students were randomly assigned seating location those seated towards the front had better attendance and learning attitudes and achieved higher grades. Interesting though, when students were switched from the front to the back halfway through the semester, those students who started at the front maintained their grades and attendance after moving to the back 6. This implies a greater role for the students’ personality, especially when behaviours have had the opportunity to establish. Current research by Losonczy‐Marshall and Marshall 10 bridges the past literature by focusing on five factors of seating choice: performance, social, asocial, noticeability, and environment. This study found students who wanted to be noticed or achieve more were seated towards the front, with those self‐selecting as asocial sitting towards the back. This provides current evidence that a student's personality traits play an important role in their choice of seating location.

This review of the literature reveals two strands of thought regarding those factors determining students seating preferences within a lecture theatre:

- Environmental factors encapsulate the ecological variables, studies relating to this are mostly concerned with how the physical space affects or influences engagement and attainment. Environmental factors include the size of the room, density, the row the student is seated on and their position on that row 3, 4, 6, 7.
- The personality of the student: It has been proposed that students who are motivated and want to engage are more likely to select a central seat in close proximity to the lecturer. Conversely, students who are less confident and do not want to engage opt for a location towards the rear of the lecture theatre to avoid the lecturer's attention 8, 9, 10.

## What are the individual environmental or personal factors directing seating choice?

There is little research to date that includes directly the opinions of the student as to why they have chosen their location within the lecture theatre. Such information is needed to guide developments in future engagement and attainment and the design of exceptional lecture spaces. This study therefore aimed to ascertain why students choose a particular location within a lecture theatre and whether their choice is a significant factor in their academic attainment. Furthermore, the research sought to discover whether student choice was congruent with staff perceptions of that choice. The research findings will be used to identify patterns in student behaviour and may help shape the way lectures are conducted.

## Methods

### Participants

The student participants were first‐ and second‐year cohorts on a range of bioscience courses including biomedical science (BMS), biochemistry (BioC), biology (Bio), and human biology (HBio). On the days of polling, ~ 154 students were present in a lecture theatre with a 254 capacity. Students were surveyed using a printed questionnaire. Separately, 23 staff members with a range of teaching experience on the same programmes were surveyed by printed questionnaires over the course of several weeks.

### Ethics

Ethics for this study were acquired following the Sheffield Hallam University Research Ethics Policy. Initial scrutiny demonstrated that no identifiable, confidential, or controversial information would be collected. No gender/age/other educational experience or other demographic factors were requested or considered within the analysis. Participation in the study was optional. Students were read and given a copy of the following statement before the collection of data, which served as a means of consent. Only the study organiser was able to determine the identity of the student sat within a given location via their student number.

‘By filling in the questionnaire you are giving consent for your location in the room to be mapped against your course, a quote of your comments and final grade (reported as a boundary e.g. 3rd, 2:2, 2:1 1st). Your name and student number will not be used in any publications. If you do not wish to take part in this study it will not effect your grades for this module’.

### Collection of data

**Figure 1:**
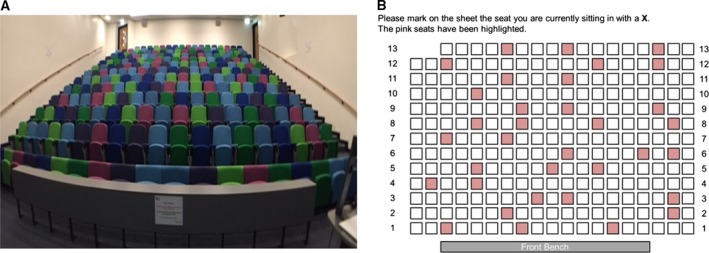
(A) The lecture theatre used in this study has 13 rows of 19 seats, with the entrance to the lecture theatre at the front left and right. The lectern is located at the bottom right of the image. (B) Students were asked to identify where they sat within the room by placing an X in the relevant box. Pink boxes were used to orientate with respect to the pink seats.

All student data were gathered from normal timetabled lectures’ sessions using the same lecture theatre (Fig. 1A) midway through a 24‐week core biochemistry module. Students self‐selected their seats before the confidentiality agreement was displayed and subsequently being handed the questionnaire. The questionnaire asked students to identify where they were seated using a grid schematic of the room (Fig. 1B) and gave a free text response to the question ‘why have you chosen to sit in this location today’. Student numbers were collected on the questionnaire to allow mapping against course of study and attainment in the coursework assessment task. Staff data were collected by an opt‐in survey, with 24 respondents covering a range of teaching experience from 1 to 35 years. Staff were asked to identify on Fig. 1B where students obtaining high, mid and low attainment would be located. An open text comment box was used to capture the perceptions on why staff thought students sat in those locations.

### Data analysis

The student and staff questionnaires were collected, and an independent researcher anonymised and transposed the comments.

Comments acquired from the three survey points were collated and mapped together within the same analysis. Student data were analysed, and each comment categorised as either F – friendship, A/V – audio‐visual reasons, I – to avoid interaction (asocial), E – to increase engagement or O – other. Categories were selected based on the phrasing used in the comments with keywords such as ‘friends’ being allocated F, ‘hear/see’ A/V or ‘engage’ E. The comment codes were then mapped onto the 16 × 9 lecture theatre grid space. The student numbers collected on the questionnaire were mapped onto seat location by the use of a 16 × 9 grid in an excel spreadsheet. This allowed individual assessment task outcomes to be mapped to individual seats by the use of a vertical lookup table. Assessments were colour‐coded by the use of three‐point conditional formatting where the lowest point was red, the mid‐point was white, and the highest point was blue. To maintain student anonymity, the actual grade values have not been reported.

Staff data regarding the perception of student seating choice were pooled and used to generate heat maps. Initially, the responses were individually analysed, for each response given, a mark of 1 was noted in an individual box on a 10 × 7 grid that corresponded to the identified seating location, for each grade boundary. Data were summed at a granular level and used to generate heat maps by conditional formatting, with white representing the lowest number of lecturers selecting that location for that grade through to red representing the highest.

### Statistics

To determine whether significant differences in attainment between friendship groups assessment marks exist, final grades were mapped onto the lecture theatre for both the first‐year discursive essay and a second‐year problem‐solving task. Group clusters were identified on the bases of course groupings that identified students as sitting within friendship groups. Statistical analysis was performed with the program StatsDirect. A Kruskal–Wallis: all pairwise comparison was performed followed by a post hoc Conover–Iman test. Significance was reported with a p value of < 0.05. To maintain student anonymity regarding the final mark acquired, data were plotted as difference to the overall class mean.

## Results

### Staff perception of student location and attainment

**Figure 2:**
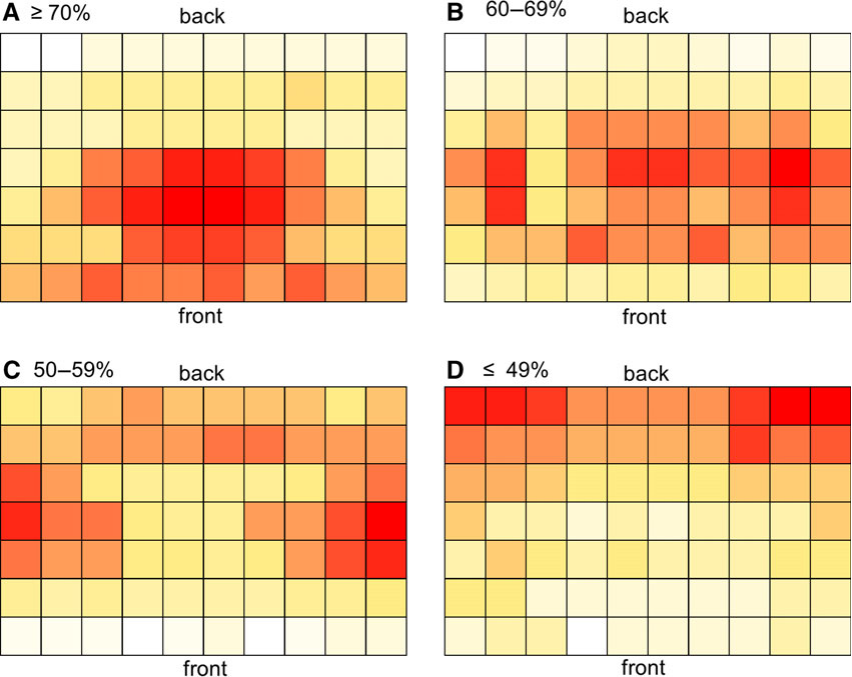
Heat maps showing lecturer perceptions of student location and level of attainment. Colour scale, white representing the lowest number of lecturers selecting that location for that grade through to red representing the highest. Perceived location of students attaining (A) ≥ 70%, (B) 69–60%, (C) 59–50% and (D) ≤ 49%.

Perkins and Wieman (2005) open their investigations into student location and attainment with the statement, ‘Every physics instructor knows that the most engaged and successful students tend to sit at the front of the class and the weakest students tend to sit at the back’ 6. To ascertain whether the perceptions of staff who teach large groups align with this assumption, 23 biosciences academic staff with a range of teaching experiences were asked to identify where they considered high‐achieving (≥ 70%), good‐achieving (69–60%), moderate‐achieving (59–50%) and low‐achieving students (≤ 49%) would locate themselves within a standard lecture theatre on a 10 × 7 grid. Data were collated, and each cell was then summed to generate heat maps indicating those areas thought to seat students gaining a given grade boundary (Fig. 2).

The heat map in Fig. 2 shows the location deemed most likely to be seating students that obtain high achievement ≥ 70% as being located in a central location close to the front of the room. This perception agrees with Perkins and Wieman's (2005) statement and results obtained by Marshall and Losonczy‐Marshall (2010) in which staff perceived students in a central location performed the best. Students achieving grades between 60 and 69% were perceived to cover a central location but are more wide spread across the rows (Fig. 2B). Students attaining a 50–59% are thought to be located near the aisles and with more frequency than higher achieving students (Fig. 2C). Students achieving a ≤ 49% were considered to occupy the back rows and towards the edges (Fig. 2D). The general trend in the heat maps shows the perception that students towards the front attain higher grades. However, the lecturers’ comments showed a more complex view of those sitting at the front, with multiple lecturers believing students who are struggling to understand the module sit on the front row.

‘students who are struggling may sit on the front to try and get more from the lecture’

‘those who struggle and want staff's attention sit on the front row’

Lecturer comments about students seated near the back are varied, with some assuming it is students who are not interested in the lecture and do not want to engage.

‘students who don't want to engage are sat at the back’

However, some comments reflect the complexity of the situation and note that although the students appear to be disengaged this is not reflected in attainment:

‘I don't equate marks with where they sit, highly competent students sit at the back along with those who would like to disengage’

‘disruptive students at the back but often very bright’

There was also a general trend in comments that presumes students who gain low attainment or fail were not present at all.

‘those that get the low marks don't attend’

‘Fail‐ No attendees’

In summary, the view of the academic staff generally supports that seen in the literature; however, there are undertones which hint to a more complex picture. There is a recognition that students’ personalities have a greater impact on their choice of location, subsequent engagement and attainment levels.

### Why students choose to sit in the location they do

Numerous studies have been performed correlating seating location with final assessment. However, very few of these studies directly investigated the student's reasons for choosing the location that they did. Often these studies report average grades per row. Here, the purpose was to identify the reasons for location choice and to assess whether patterns arise in the final location within the classroom. To achieve this, first‐ and second‐year students undertaking bioscience‐related degree programmes where polled in an opt‐in survey during core biochemistry modules. Response rates of 86% from 154 first‐year students and 55% from 151 students in the second‐year present on the day of polling were obtained. Students were asked to identify the seat they were sitting in and record their student number, and this allowed attainment in assessment tasks and course identity to be mapped to location. The students were also asked to comment directly on ‘Why are you sitting in the location you are today?*’*. Comments were blinded and categorised into groups (Table 1).

**Table 1:** Key showing the abbreviations used to group comments for analysis. Comments were blinded and coded into each category. An example comment is given in each case

Code	Class	Comment example	Frequency
F	Friendship	‘this is where my colleagues sit’	127
A/V	Audio‐visual reasons	‘see without straining’	151
I	To avoid interaction	‘don't have to interact with the lecturer’	9
E	To increase engagement	‘feel more engaged with the lecture’	9
O	Other	‘I like being at ends to escape’	43

### Students are located in course groups with friends

**Figure 3:**
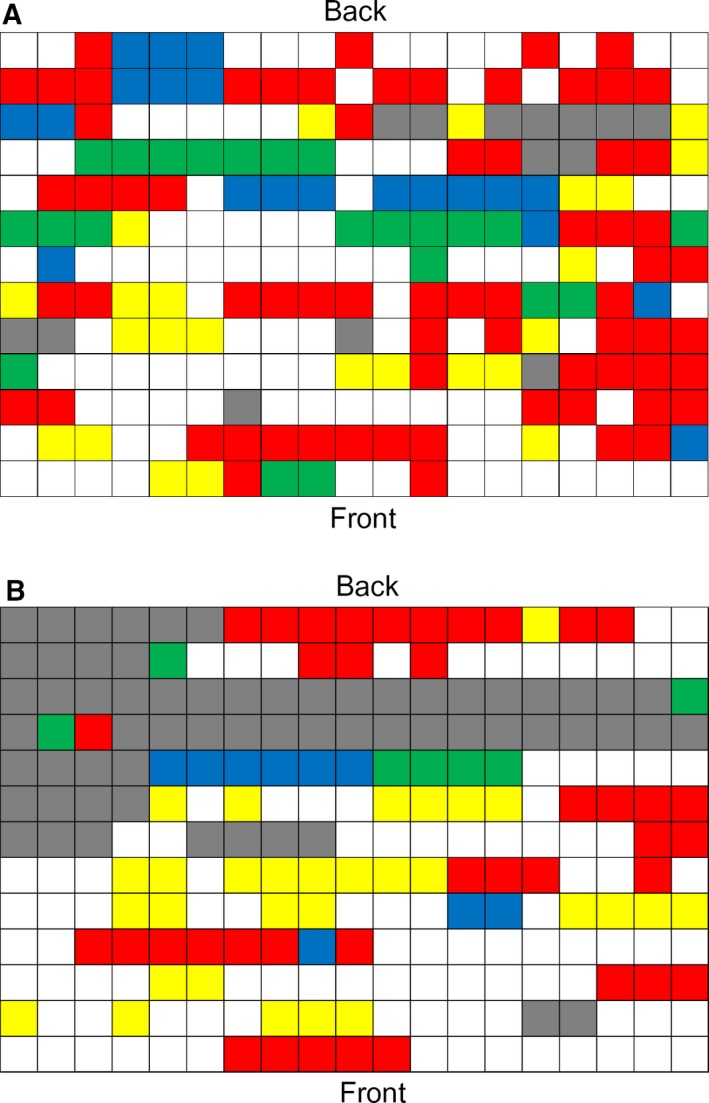
It shows the seating location of participating students. Students were from the following course: biomedical science (red), biology (green), biochemistry (blue) and human biology (yellow). Grey areas represent students that were present and chose not to participate. (A) First‐year students and (B) second‐year students.

The data on the student's location within the room were plotted using student number as a place holder. The course the students are enrolled on was colour‐coded and highlighted on the map of the lecture theatre (Fig. 3). Collation of student questionnaire responses showed students tend to form clusters with others on the same course. These clusters were in small rows of three to seven students. The majority of students who declined to take part in the survey were located towards the back (rows 10 to 11 indicated in grey in Fig. 3), but not directly at the back of the lecture theatre (row 13). There was no clear pattern for each course sitting within a given location in the room showing there was no inherent preference. Some clusters within the first‐year data contained multiple courses within a group; in these instances, these clusters reflected peer groups who had progressed from a foundation degree programme and had chosen to sit as established friendship groups.

‘we as ex‐foundation year all sit together, because we are friends’

‘Sat with friends from foundation year’

**Figure 4:**
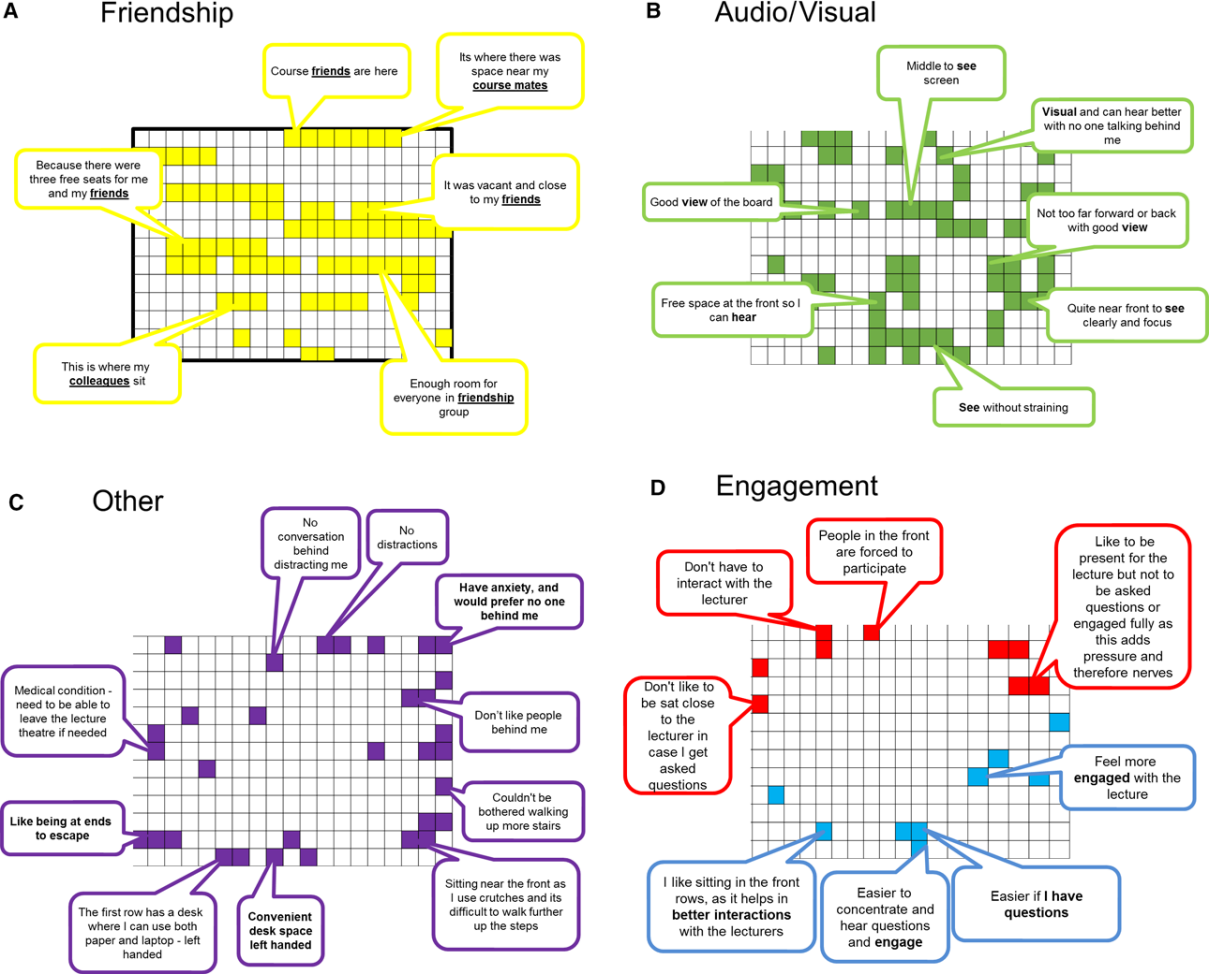
Comment codes were collated, aggregated for the survey points and mapped onto the location in the lecture theatre. (A) Friendship comments, (B) audio‐visual reasons, (C) other and (D) engagement.

To identify how student comments related to the lecture environment, data were combined from both first‐ and second‐year students and plotted onto seat location (Fig. 4). Analysis of the comments demonstrates that both first‐ and second‐year students choose their seating location predominantly to be able to sit with friends (Table 1, Fig. 4A). No inherent pattern was observed to explain cluster location, but often one member of the group would give a reason other than friendship for sitting in a given location, with their friends then sitting with them (Fig. 4A).

Many students also said they sat in their chosen location for the best visual and auditory experience, although their views on what was best varied. Some students said they found the back‐distracting and noisy and opted for a place closer to the front, while others said the back was the best place for them to see and concentrate without distraction (Fig. 4B). After receiving these contradictory comments, noise levels were recorded at the back row, middle row and front rows during a lecture. There was no difference between the average decibels on the back row (67.7 dB) and the front row (67.5 dB). These comments would indicate that the reasons for picking a location with the lecture theatre based on ability to see or hear are personally subjective.

Comments classified in the ‘other*’* category appear to ring the edges of the lecture theatre. Students who choose the front row revealed that practical reasons influenced their decision, for example being short‐sighted, being left‐handed or using a laptop. The lecture theatre used in the study was fitted with a single bench across the front row. In all other locations, tables folded out from the right. Only the middle of the front row provides a solid desk surface suitable for left‐handed people with room to comfortably use a laptop. The pull‐out desks on the other rows provide less support for left‐handed people to lean on and write. For these students, the reason for their seating locations was ecological, demonstrating that the design of the lecture environment influenced the decision to sit in a front central location. The edges of the room near to the staircases had clusters of comments stating that students found these locations ‘safe’. Students chose these locations to reduce anxiety or to be ‘able to escape’ or because they did not want to have people sat behind them. A few students located at the back also shared this view (Fig. 4C).

Students who identify with wanting to engage directly with the lecture and feel involved with the session are seen seated in central locations at front of the lecture theatre. These students are then sitting in an area that allows direct interactions with the lecturer. Conversely, students towards the back of the room identify with not wanting to interact with the lecturer (Fig. 4D). These locations correlate with staff perceptions of attainment and would strengthen the view that students who interact are perceived to achieve more highly. In summary, students have varied contradictory and complex reasons for sitting in particular locations, and as such, any attempt to move them should be carefully considered.

### Friendship groups obtain similar grades in problem‐solving tasks

**Figure 5:**
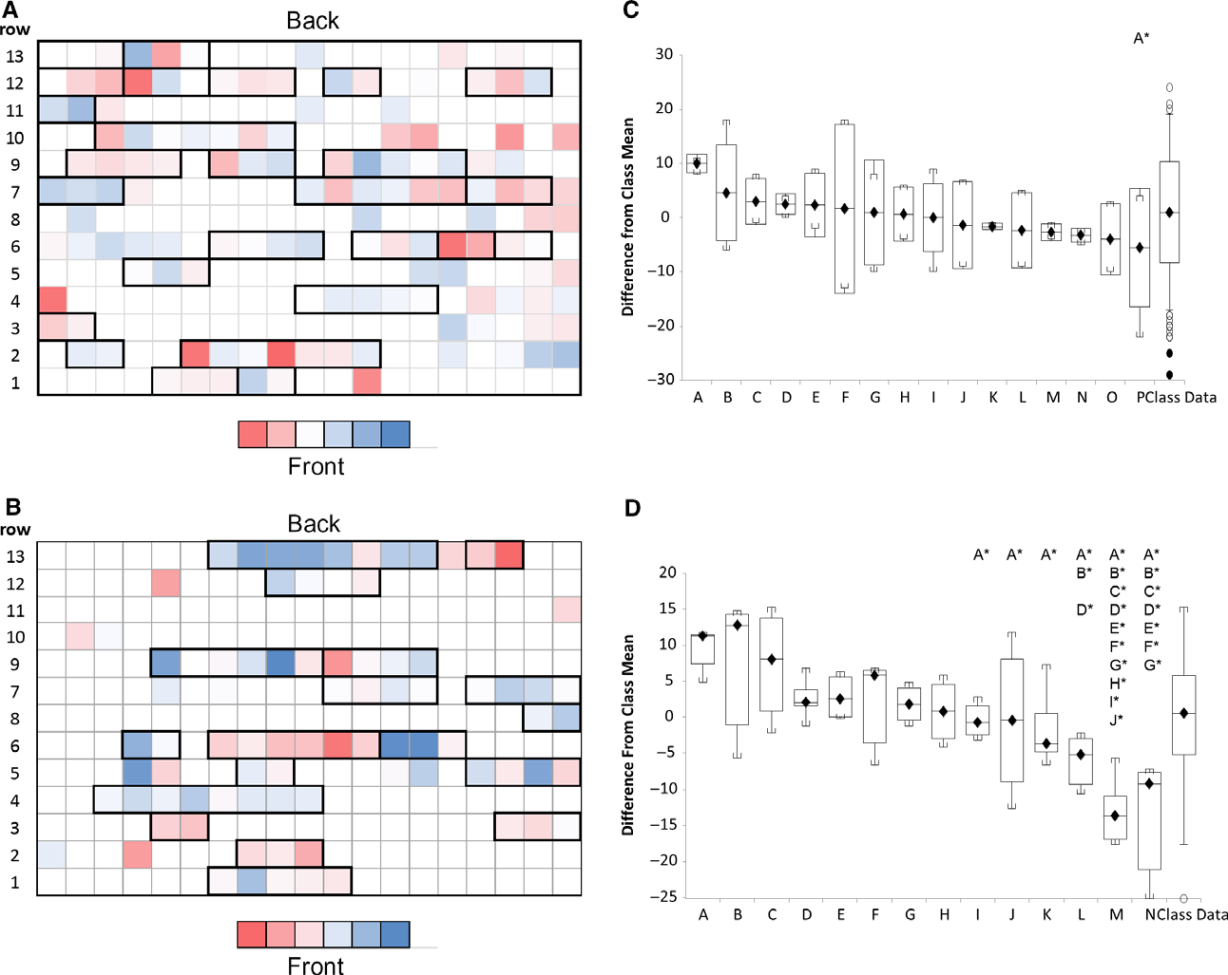
Attainment grouping. Individual attainment in either (A) a first‐year essay task or (B) a second‐year problem‐solving task was mapped onto the lecture space. Assessments were colour‐coded by the use of three‐point conditional formatting where the lowest point was red, the mid‐point was white, and the highest point was blue. Individual course groupings were identified from the student response and are highlighted as thick black boxes on the diagram. (C) and (D) show the individual mark difference from the group mean for each of the course groupings identified as box and whisker plots with mean, standard deviation and range noted. Significance was determined by Kruskal–Wallis: all pairwise comparison followed by a post hoc Conover–Iman test. Significance was reported with a p value of > 0.05 and is indicated on the figure by X* where X referees to the comparisons group.

Having identified that students self‐select locations alongside peers, the research aimed to look for a correlation between seating choice and attainment. Assessment marks were mapped onto the lecture theatre for both the first‐year discursive essay and a second‐year problem‐solving task (Fig. 5). The first‐year task was an individual discursive essay of 500 words, submitted midway through the module (Fig. 5A). The second‐year students were given a problem‐solving exercise that required the application of knowledge presented during the lecture series, submitted as a formative assessment four times through the module. This was supported with in‐class problem‐solving sessions in which peer‐to‐peer collaboration was central (Fig. 5B).

The average score for each row was determined; in the case of rows 10 and 11 in Fig. 5B, this area contained students known to be present but who chose not to respond. The average score for each row showed no clear correlation or pattern of marks, with students located towards the back equally as likely to score the same mark on average as those towards the front. Further analysis of the data in Fig. 5B showed that in the case of the second‐year problem‐solving assessment, the marks clustered into groups. When the course location information was added to the data shown here as bold outlines, it can be seen that students seated on the same row and on the same course were obtaining similar marks. This is particularly evident for row 6, Fig. 5B, in which a high‐achieving group is seated next to a lower achieving group. This patterning was not observed for the first‐year student group in their individual essay task. Fig. 5C,D shows the individual attainment for the course groupings as box and whisker plots. To maintain student anonymity regarding the final mark, acquired data were plotted as difference to the overall class mean and organised from high to low attainment. Figure 5C shows the spread of attainment for the friendship groups resulting from an individual essay assessment task. The only significant difference observed was between a high‐attaining group of three students (A) and a cluster of lower attained groups (M through P). Conversely, a clear spread in attainment between the friendship groups can be observed within problem‐solving assessment task as shown in Fig. 5D. Significant differences were observed between the high‐ and low‐attainment groups, with group A significantly outperforming groups I through N and group B outperforming groups L through N. Groups C to G when compared to groups M and N also showed significant differences in average mark. These data would indicate that within the problem‐solving task, the make‐up of the friendship group has a significant impact on final attainment.

## Discussion

This study aimed to find out why students choose particular locations within a lecture theatre and whether their choice is a significant factor in their academic attainment. Further, the research sought to discover whether student choice was congruent with staff perceptions of that choice. Student comments specifically around engagement and anxiety do show clustering to specific areas of the lecture theatre (Fig. 4). Those wishing to engage with staff where seated towards the front, whereas those who indicated that they did not want to engage were seated at the back. This pattern correlated with staff perception of attainment and would indicate that staff correlate overt engagement with higher achievement. However, no direct correlation between location and attainment was observed. One issue identified with large classes is that social processes can start breaking down. Students can become alienated, and antisocial behaviour at the back of lecture theatres can proliferate 11. This can manifest itself as those students at the back of the room being perceived not to be paying attention, as highlighted by the staff comments and the students’ comments on not wishing to engage. What is clear both from the literature and from this study is those students who want to engage with the lecturer are seated near the front and those who do not are seated towards the back 10, 12.

### Peer interactions and peer learning

This work has identified the main reasons for being located within a discrete area of the room is to be sat within friendship groups. This manifests itself as small groups of three to seven students from the same course sat in rows throughout the lecture theatre. These course group clusters typically then obtain similar marks on problem‐solving assessments (Fig. 5). It is well reported that interaction with peers can positively influence overall academic development, knowledge acquisition, analytical and problem‐solving skills, alongside self‐esteem 13, 14, 15. These peer groups can act as a reference point for norms during the person's educational experience, influencing attitudes to attainment, achievement and further aspirations 16. However as seen in Fig. 5D, some self‐selected peer groups are low achieving, which raises the difficult question of the impact peer interactions with academically weak friendship groups can have. Interactions within these groups may well reinforce misunderstanding or validate negative learning attitudes and only be picked up by the tutor at the assessment stage. Such peer effects occur when a person's behaviour is affected by their interaction with peers who are ‘equals’. In the higher education setting, these effects are an emergent property brought about by the interactions between students 17. Peer influence can have a strong effect on work ethic. If my friend works hard, I feel compelled to work hard as well. If my friend does the minimum to pass, I will also do the minimum to pass. The educational goals will be set by the group and might not be aligned to the learning outcomes set by the tutor. A study by Akhtar *et al*. identified three main trends in group formation: (a) peers from a similar cultural background, most importantly sharing the same language; (b) peers who shared their social events; and (c) peer groups formed on the basis of perceived similar intellectual levels. In addition, the interactions between these groups can be defined as follows: (a) information peer – communication is focused on the exchange of information; (b) collegial peer – acts as workplace‐based friend; and (c) special peer – friend outside the workplace 18. Within the classroom setting, both collegial and special peers are considered friends. Students with special and collegial peer relationships disclose more and provide more social support to one another than students with information peer relationships 19. It is these special and collegial peer relationships interactions that are proposed to be influencing attainment in this study 20. Peer groups and their interactions are a key part of a student's experience and attainment, and it is therefore unlikely that the groups observed within the lecture theatre here are based on chance encounters and instead are likely to have been forged through and operate within these criteria. Understanding how and when these peer groups form and how they interact within a taught session is then critical in gaining the best learning outcomes for the whole student cohort.

### The anxious or nonengaging student

This study has shown no correlation between staff perceptions of engagement and students’ attainment (Figs 2 and 5). Within the group of staff polled, the predominant perception of student location was that high‐achieving students were located in the front centre of the lecture theatre, with low‐achieving students towards the back (Fig. 1) which correlates with much of the published literature 3, 4, 6, 10. Several students commented that the seat locations towards the front allowed them to interact more positively within the lecture theatre environment, in the same area identified by staff as seating high achievers. Students who do not want to directly engage with a lecturer identified as being seated at the back of the lecture theatre. However, there was no direct evidence of an impact on attainment (Fig. 5). This study then correlates with that of Kalinowski and Taper 5 and found no direct link between student location in the lecture theatre and attainment (Fig. 5).

Students whose reasons for their seating choice were classified as ‘O’ other tended to ring the lecture theatre. Many of these students identified as being anxious and chose their seat to prevent people sitting behind them, or sat at the sides to better manage their anxiety (Fig. 5C). This leads to the hypothesis that the lecture theatre is viewed as an inherently alien space for these students, yet coping strategies for ensuring they get the most from it have emerged. The reasons for a student not wishing to engage with the lecturer are stated in this study as ‘nerves’ and ‘not to be asked questions’ (Fig. 5D). In terms of attainment, social anxiety manifesting as a lack of interaction had a significant and negative relationship with academic achievement. Here, these isolated students typically scored lower than the group average as shown by the red boxes in Fig. 5. This highlights the critical role that social ties appear to play in successful academic outcomes and the positive effects of alleviating social anxiety during study 21.

### Recommendations

The peer groups in this study with lower attainment are a challenge to peer learning and group work interventions. The risk is that misunderstanding or self‐validation of incorrect ideas can occur and be propagated through the group leading to low marks. These incorrect ideas are then only picked up by the tutor at the assessment stage.

Recommendation 1: Breaking up lower attainment peer groups by creating random or mixed ability groups. This is not only a high staff‐input solution but is likely to be detrimental to group and course identity once groups are formed, as group membership converts from special peer relationships to information peers, which has been shown to decrease engagement 20. As such, this approach should be used sparingly. It would be more beneficial than to create an environment in which mixed ability groups can form from the outset, by encouraging interactions between students during the early induction phase of their course.

Recommendation 2: During think–pair–share interactions, rather than asking the students to talk with the person next to them, as advocated by King 22, ask the students to talk with people in front or behind them that they may not know. Formative tasks can be performed with students outside their established friendship group by using other surrounding students, brief conversations can be conducted or work exchanged. These transient interactions with information peers may well lead to a broader exchange of knowledge and understanding, without the need to establish a new working relationship 23.

Recommendation 3: Targeted intervention within the classroom can also occur through the use of student response systems for key learning points. This may well help identify areas of misunderstanding for the student who would rather not engage openly 24, 25. Such systems allow students who otherwise would not verbally contribute to the teaching session to interact and assess their own learning.

## Conclusion

What is evident from this study is that students choose to sit where they are comfortable, either physically, mentally or socially, and this needs to be respected. The range of learning spaces that a student may find themselves in is vast. It is not suggested that the exact patterns observed here will replicate in any given environment; rather, the pattern of student location will be driven by the desire to sit within friendship groups and/or where the student feels comfortable, be that for reasons of audio‐visual requirements or psychological safety. If someone has sat at the back to avoid anxiety brought about by direct interaction, or for a clear view of the screen, forcing these people to move to the front may not be a benefit for their engagement and alternative means of interaction should be explored. Allowing students to sit where they choose, the use of activities that enable nonthreatening interactions with the tutor and a means of self‐checking within diverse peer groups may well result in increased engagement with the material.

## Author contributions

DPS conceived and designed the project, AH and DPS acquired the data, DPS and ML analysed and interpreted the data, and DPS, AH and ML wrote the manuscript.
